# Patterns and Challenges in Help-Seeking for Addiction among Men: A Systematic Review

**DOI:** 10.3390/jcm13206086

**Published:** 2024-10-12

**Authors:** Julio A. Camacho-Ruiz, Carmen M. Galvez-Sánchez, Federica Galli, Rosa M. Limiñana Gras

**Affiliations:** 1 Foundation Project Man Jaén, 23002 Jaén, Spain; julioangel.camachor@um.es; 2 Department of Personality, Evaluation and Psychological Treatment, Faculty of Psychology and Speech Therapy, University of Murcia, Building 31, 30100 Murcia, Spain; liminana@um.es; 3 Regional International Campus of Excellence (CEIR) Mare Nostrum Campus (CMN), 30100 Murcia, Spain; 4 Department of Dynamic and Clinical Psychology, and Health Studies, Faculty of Medicine and Psychology, SAPIENZA University of Rome, 00185 Rome, Italy; f.galli@uniroma1.it; 5 Assisted Reproduction Unit, QuironSalud Murcia Medical Center, 30008 Murcia, Spain

**Keywords:** addiction, help-seeking, men, barriers, facilitators, gender perspective

## Abstract

**Background/Objectives**: Addictive and substance-related disorders represent a substantial public health challenge, marked by rising incidence and prevalence rates. Men and women exhibit different patterns of help-seeking for health and social issues including addictions. This research aimed to analyze the help-seeking process among men with addiction to improve understanding and develop more effective, person-centered treatments. **Methods**: This systematic review was performed based on the Cochrane Collaboration guidelines and reported following the Preferred Reporting Items for Systematic Reviews and Meta-Analyses (PRISMA) guidelines. The protocol of the revision was registered in advance in PROSPERO. Searches were conducted in the PubMed, Scopus, and Web of Science (WOS) databases. **Results:** Based on the literature search, 16 studies were included in the current systematic review. The analyzed studies included seven on substance-use disorders, three on gambling disorder, two on tobacco-use disorder, two addressing substance-related disorders in general, one on opioid-use disorder, and one focused on marijuana use. Gender differences influenced help-seeking behavior, with women generally exhibiting a higher propensity to seek assistance for addiction-related issues than men. Seeking help for addiction—whether substance use or gambling—can be hindered by several barriers, particularly public stigma and discrimination, which tend to be more pronounced for alcohol and gambling compared to tobacco. Additional barriers in the help-seeking process include negative beliefs and attitudes toward seeking help, often associated with traditional male gender roles. Each substance-use disorder was analyzed in depth to gain a better understanding of the barriers faced by this population. **Conclusions**: Integrating a gender perspective into the diagnosis, prevention, and treatment of addiction is essential. As addiction patterns vary between men and women, approaches must be tailored accordingly. Recognizing men as a distinct group in research and clinical practice is also crucial for developing more effective and personalized treatments.

## 1. Introduction

Addictive and substance-related disorders constitute a significant public health challenge, with increasing incidence and prevalence [1]. Each year, over three million people, primarily men, die due to alcohol and drug use [1]. A recent report by the World Health Organization (WHO) revealed that there are 2.6 million alcohol-attributable deaths annually, representing 4.7% of all deaths, and 0.6 million deaths due to psychoactive substance use. In particular, two million of these alcohol-related deaths and 0.4 million of the psychoactive substance-related deaths were men [1]. The United Nations Office on Drugs and Crime (UNODC) underscores that the adverse impacts of the global drug problem are intensifying amid the expansion of drug consumption and markets [2]. Although approximately 64 million people worldwide suffer from drug-use disorders, only one in 11 receives treatment [2]. Women have even less access to treatment than men: only one in 18 women with drug-use disorders receives treatment, compared to one in seven men [2]. In addition, people with substance-use disorders are six times more likely to die by suicide in comparison with the general population, and men with substance use issues are three times more likely to commit suicide than men without such problems [3].

The Diagnostic and Statistical Manual of Mental Disorders, Fifth Edition, Text Revision (DSM-5-TR) includes an extensive section focusing on addictive and substance-related disorders. The category of addictive and substance-related disorders encompasses the following: alcohol-related disorders, caffeine-related disorders, cannabis-related disorders, hallucinogen-related disorders, inhalant-related disorders, opioid-related disorders, disorders related to sedatives, hypnotics, or anxiolytics, stimulant-related disorders, tobacco-related disorders, disorders related to other substances (or unknown substances), and non-substance-related disorders, which include gambling disorder [4].

On one hand, the fundamental characteristic of a substance-use disorder is a collection of cognitive, behavioral, and physiological symptoms that indicate continued substance use despite significant related issues. One relevant characteristic of substance-use disorders is the changes in neural pathways, which may continue beyond the detoxification phase, particularly in individuals with more severe conditions. These brain changes can lead to behavioral manifestations such as repeated relapses and intense drug cravings when individuals encounter drug-related cues. Moreover, the pathological pattern of behaviors associated with substance use is at the base of its diagnosis [4].

On the other hand, gambling implies risking something of value with the expectation of gaining something of greater worth. Different people across several cultures participate in gambling activities, most of whom do so without showing trouble. Nevertheless, some people experience significant impairments related to their gambling behavior. The defining characteristic of gambling disorder is persistent and recurrent maladaptive gambling that interferes with personal, familial, and/or occupational functions. Research suggests that gambling behaviors provoke the activation of reward systems in a similar way to those stimulated by addictive substances, resulting in behavioral symptoms related to those found in substance-use disorders [4].

Men and women exhibit differing patterns of help-seeking for health and social issues including addictions [5]. Men who conform to hegemonic or dominant masculinity stereotypes often demonstrate a reluctance to seek help or consult healthcare professionals. This reluctance is especially pronounced in the context of mental health issues such as depression or addictions. Frequently, drug use is utilized as a way of self-medication to answer episodes of depression or anxiety. Within the framework of traditional or hegemonic masculinity, seeking help is often perceived as a sign of weakness or vulnerability, particularly in light of the prevalent gender stereotypes associated with this construct of masculinity such as “being strong” or “being tough”. In contrast, traditional femininity promotes the notion that women should be delicate, vulnerable, and more prone to seeking help [5,6,7].

Moreover, for people experiencing addiction (i.e., alcohol, gambling, drug abuse, etc.), perceptions of stigma may adversely influence both the decision to seek help and the timing of when individuals do so [8,9,10].

Public stigma is characterized by negative perceptions and stereotypes held by the majority toward a specific group [11]. The perception of difference is a core component of stigma and helps us understand how the affected group is considered distinct from the majority [12]. This sense of differentness can be conceptualized as a shared stereotype across various stigmatized conditions. Furthermore, for stigma to manifest, this perception of differentness must evoke disdain, rather than merely signify a neutral difference [13]. In addition, a process known as self-stigma can occur when individuals within a stigmatized group begin to internalize the public stigma directed at them.

Women are generally more inclined to seek assistance for gambling-related issues [14]. Rockloff and Schofield [15] observed notable gender differences in the likelihood of seeking help for problem gambling. Men are often less willing to seek help due to feelings of shame, embarrassment, and the stigma attached to it. In contrast, women tend to postpone seeking help, frequently due to denial of the problem or hesitation to quit gambling, as it may lead to the loss of a significant social support network. In the instance of alcohol-use disorder, women appear to be less affected by social stigma compared to men and tend to seek help more frequently than men [16]. In the context of tobacco, male gender norms appear to discourage men from seeking help, while gender stereotypes also affect women by increasing the negative connotations associated with tobacco use in women [17].

A key implication of these findings for psychologists and the broader health and social care professions is the necessity of integrating a gender perspective and developing gender-sensitive intervention programs specifically tailored to address addictions within the male population. It is relevant to acknowledge that men are rarely the focus of interventions employing a gender perspective [18], and this perspective is seldom applied to psychological interventions involving men. This gap arises because interventions often use the androcentric model of the White, Catholic, heterosexual, middle-class, middle-aged man as the normative framework for addressing issues in other groups [18]. Consequently, in psychological interventions, particularly in addiction treatment, it is essential to consider the gender norms enforced by patriarchy when working with men.

Our patriarchal system is fundamentally responsible for excluding men from emotional well-being as it often compels them to engage in actions essential for developing traditionally masculine behaviors related to control and dominance over others, such as violence, authoritarianism, and competitiveness, behaviors that, in most cases, entail the suppression of empathy. Thus, the control and dominance that may initially appear to be privileges granted by patriarchy conceal significant suffering. This disconnection from emotional well-being has frequently led men to develop violent behaviors, refrain from seeking help for health-related issues due to adherence to gender norms that discourage vulnerability, and engage in substance abuse, which persists in the personal and social lives of many men. This existential gap is often referred to as a crisis of masculinity; however, it is more accurately described as a crisis of patriarchy. Therefore, it is crucial to recognize that patriarchy itself undermines masculinity, hindering men from experiencing essential emotional aspects necessary for personal well-being, which all individuals seek to facilitate change, thus complicating the processes of help-seeking [19]. Consequently, the implementation of gender-sensitive interventions could assist men with addiction issues in improving their emotional well-being and managing their emotions more effectively as well as in developing a more proactive approach to help-seeking.

To the best of our knowledge, this is the first systematic review to examine the process of help-seeking among men with addiction. Based on previous findings, the primary objective of this research was to analyze the help-seeking process in men with addiction, with the aim of contributing to a better understanding of this phenomenon, and in turn, facilitating the design of more effective and person-centered treatments. The specific objectives were as follows: to analyze the help-seeking process from a gender perspective, identify the main barriers and facilitators in the help-seeking process among men with addiction, and identify potential research gaps in the field.

## 2. Materials and Methods

### 2.1. Search Strategy

The current research was performed following the guidelines of the Cochrane Collaboration and was reported in alignment with the Preferred Reporting Items for Systematic Reviews and Meta-Analyses (PRISMA) [20]. The review protocol was previously registered in the international database PROSPERO: Prospective Register of Systematic Reviews [registration ID: CRD42024580244]. The search terms utilized included “men”, “help-seeking”, and “addiction”, which were derived from Medical Subject Headings (MeSH). The following search string was used in PubMed [“men” and “help-seeking” and “addiction”]; SCOPUS: [“men” AND “help-seeking” AND “addiction”], and WOS [“men” and “help-seeking and “addiction”]. The search strings were used with two main filters: one to restrict the search to the last ten years, and the other to include only papers written in English. The PICO question guiding this review was: What is the process of help-seeking among men with addiction?

**Figure 1:**
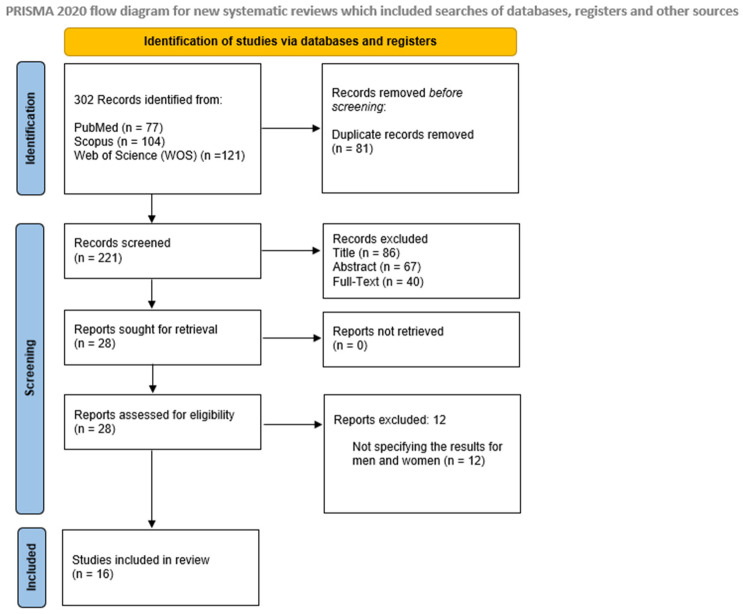
Flow diagram of the process of help-seeking among men with addiction.

Searches in the PubMed, Scopus, and Web of Science (WOS) databases were conducted by all researchers (J.A.C-R., C.M.G-S., F.G., R.M.L.G). Articles were first screened by examining their titles and abstracts to eliminate irrelevant studies. The remaining articles were then thoroughly reviewed for eligibility. Full texts of the pertinent studies were subsequently obtained and evaluated according to the inclusion and exclusion criteria, leading to the final selection of articles for review. The PRISMA flowchart (Figure 1) shows the different steps in the screening and selection process. The final search was completed on 31 August 2024.

### 2.2. Eligibility Criteria

The following inclusion criteria were used: (1) peer-reviewed original research on help-seeking processes in men with addiction including several study designs (e.g., qualitative, quantitative, and/or mixed methods); (2) involved adult male participants aged 18 years or older who were diagnosed with an addiction; (3) were published within the last ten years (2014–2024); and (4) were published in English. Exclusion criteria were: (1) review articles or meta-analyses; (2) comments, editorials, case reports, letters, or meeting/conference abstracts; and (3) duplicated studies.

### 2.3. Data Extraction and Quality Assessment

Firstly, the characteristics, methodologies, and findings of the studies were extracted along with the addiction-related issues. These details are summarized in Table 1.

The quality assessment was performed independently by two researchers (J.A.C-R. and C.M.G-S.) with an initial agreement rate of 95%. To reach a consensus, any disagreements were discussed with a third reviewer (R.M.L.G.). This quality assessment focused on analyzing the limitations of the selected studies and identifying potential risks of bias. The quality of the studies and the risk of bias were evaluated using the Effective Public Health Practice Project Quality Assessment Tool (EPHPP) [21]. This tool includes six dimensions: Selection Bias, Study Design, Confounders, Blinding, Data Collection Method, and Withdrawals and Dropouts. Each dimension was rated as strong (score of 3), moderate (score of 2), or weak (score of 1). Additionally, the overall quality of each article was categorized as follows: 1 = Strong (no weak ratings), 2 = Moderate (one weak rating), or 3 = Weak (two or more weak ratings).

### 2.4. Data Synthesis

In alignment with the main objective of this systematic review, the authors evaluated the primary objectives, methodologies, and the presence or absence of control groups in each study. Additionally, the clinical implications of the key findings and the major limitations of each study were assessed (see Table 1 for further details). Particularly, each author analyzed the help-seeking process from a gender perspective including both men and women, although the focus of the research was on men. The information about women was included solely for descriptive and comparative purposes. The barriers and facilitators in the help-seeking process among men with addiction were examined in each study, highlighting the main similarities and differences. Additionally, each study was analyzed to identify potential research gaps in the fields of research and intervention, contributing to improvements in these areas and increasing the scientific evidence related to the topic. First, all authors conducted a preliminary analysis individually, focusing on the characteristics of the help-seeking process from a gender perspective including both men and women with a particular emphasis on men’s patterns. Subsequently, they performed a similar analysis to identify the barriers and facilitators in the help-seeking process among men with addiction and to determine potential research gaps in the fields of research and intervention. In the second stage, the authors shared their analyses and discussed the relevance of their findings based on the previous literature and from a gender perspective. The quality and the risk of bias for each study were also analyzed and reported in the Quality of Selected Studies and Risk of Bias section and Table 2. These analyses were performed to analyze the main characteristics of the help-seeking process among men with addiction, with the aim of enhancing clinical interventions and guiding future research in this field. Any discrepancies identified during the article review process were addressed by consulting the senior author (R.M.L.G.).

## 3. Results

### 3.1. Literature Search and Study Characteristics

A total of 302 articles were initially identified from all database searches, of which 221 were chosen for screening. The detailed inclusion process is illustrated in the PRISMA flowchart (Figure 1). At the end, 28 full-text articles were analyzed for eligibility in this systematic review. Of these, only 16 met the inclusion criteria and were thus included in the data extraction (Table 1) and quality assessment processes (Table 2).

The publication of the selected studies took place between 2014 and 2024. At the methodological level, eight studies were qualitative designs [22,23,24,25,26,27,28,29]; five were cross-sectional [30,31,32,33,34], two were retrospective designs [35,36]; and one was based on concept mapping: a mixed methods approach to participatory research [37].

Regarding the countries of each study, four studies were developed in United States of America (USA) [24,31,32,33], two in Sweden [23,36], two in India [25,30], one in Qatar [22], one in China [26], one in Iran [29], one in Canada [37], one in Denmark [34], one in the United Kingdom [27], one in Australia [35], and one in Thailand [28]. The 16 selected studies included a total of 18,795 participants (age range: 18–70 years), of which 1645 were women and 2418 were men. In some studies, only the percentage of each gender was provided, making it impossible to determine the exact number of participants for each gender (see Quality of Selected Studies and Risk of Bias section for further details). Based on our objectives and the selected studies, the Results section addresses three main issues: the help-seeking process from a gender perspective, the primary barriers and facilitators in the help-seeking process among men with addiction, and potential research gaps in the field. Although women were analyzed for descriptive or comparative purposes in some cases, the focus of this paper was on men with addiction.

#### 3.1.1. Help-Seeking Behaviors Related to Addiction

The addictions analyzed in the studies were seven studies about use disorder [23,24,25,27,28,31,34], three about gambling disorder [35,36,37], two about tobacco disorder [22,26], two about substance-related disorders in general [29,33], one about opioid-use disorder [30], and one study about marijuana [32] (see Figure 1 and Table 1 for more details).

The studies reviewed suggest that help-seeking behavior in the context of addiction—encompassing substance use (such as alcohol, tobacco, opioids, and marijuana) as well as gambling—might be adversely affected by public stigma and discrimination [22,23,25,34,37]. Furthermore, women generally show a greater tendency to seek help for issues related to addiction including gambling [36] and alcohol-use disorder [27]. In contrast, men are often less likely to seek assistance for addictions (such as gambling and alcohol-use disorder, among others) due to feelings of embarrassment, shame, and the associated stigma, along with other potential factors [27,30,37].

#### 3.1.2. Key Barriers and Facilitators in Help-Seeking among Men with Addiction Issues

Regarding the main barriers to seeking help among men with addiction problems, the analyzed studies highlight public stigma [22,27,34], self-stigmatization [28], shame and low self-esteem [28], norms of hegemonic masculinity [22,26], and a lack of information and/or knowledge about available resources for receiving help and the processes for accessing them [27,28,30]. In contrast, as facilitators of help-seeking, the authors indicate that social support is one of the primary factors for individuals with addiction [34]. Additionally, the potential use of social networks can promote the increased utilization of addiction clinics and enhance help-seeking behaviors among individuals with addiction [30].

#### 3.1.3. Current Gaps in Research and Clinical Practice

Related to the gaps in research and clinical practice, the studies concur on the necessity of implementing interventions to reduce the stigma associated with addictions and to address the comorbidities present in these individuals [25,31,34,35]. Furthermore, a deeper understanding of the barriers in the help-seeking process is essential for developing more effective strategies to encourage men to seek help [22,23,25,30,34,37] (see Figure 1 and Table 1 for more details).

**Table 1:** Characteristics of the relevant eligible studies regarding the process of help-seeking among men with addiction.

First Author (Publication Year), Study Name, Country	Objectives	Study Design/Procedure	Sample Size (Age)	Classification of Addiction	Instruments and Variables	Results
**Al-Jindi et al. (2024). Barriers to seeking tobacco cessation services: a qualitative assessment of university students’ needs in Qatar. Qatar [22].**	To investigate the factors related to tobacco use and the pursuit of cessation services among university students.	Qualitative design. Semi-structured interview.	20 individuals: 16 men and 4 women (age range: 20–31 years).	Tobacco disorder.	Semi-structured interview (including tobacco use patterns such as the types of tobacco products used in the past or currently, the age at which consumption began, the frequency and contexts of current use, previous attempts to quit smoking, and the resources utilized in those efforts, among other factors).	Barriers to accessing tobacco cessation services: misunderstandings regarding nicotine-related problems, unfavorable views on the effectiveness of cessation programs, self-efficacy in quitting without professional help, and concerns or preferences related to the attributes of practitioners at clinics; a male-dominated culture that deters men from seeking help for cessation; societal stigma related to women’s tobacco use in Arab culture; and limited time available to attend addiction clinics.
**Jirwe et al. (2024). Alcohol Dependence, Treatment Seeking, and Treatment Preferences Among Elderly: A Qualitative Study. Sweden [23].**	To examine the viewpoints of older adults regarding alcohol dependence, their behaviors in seeking treatment, and their preferences for treatment, as well as to investigate potential gender differences.	Qualitative design. Focus group (2) interviews and individual semi-structured interviews (10).	13 individuals: 8 men (mean age of 72 years) and 5 women (mean age of 70 years).	Alcohol-use disorder.	Focus group interviews and individual semi-structured interviews. Additional information: AUDIT.	The majority of men had not formerly sought treatment for their alcohol problem, while all the women had. Women reported experiencing alcohol-related issues for a longer duration than men.
**Robles et al. (2024). Men of Mexican ethnicity, alcohol use, and help-seeking: “I can quit on my own”. United States of America (USA) [24].**	To investigate help-seeking behaviors among Hispanic men of Mexican descent and to gain a deeper understanding of how they express their decision-making process when seeking assistance.	Qualitative design. In-depth semi-structured interviews.	27 men (mean age = 35.7, SD = 10.82). SD: standard deviation.	Alcohol-use disorder.	Screening: BAC and AUDIT. In-depth semi-structured interviews focused on the help-seeking process.	Men indicated that their experiences with seeking help were frequently preceded by adverse social consequences and described their overall experiences with help-seeking as negative. Barriers identified in the help-seeking process included: negative beliefs and attitudes toward seeking help. Perceptions of treatment as a financial rather than a personal issue, a lack of respect or understanding from professionals, and the presence of stigma among these professionals.
**Wallhed Finn et al. (2023). The associations between public stigma and support for others’ help-seeking for alcohol-use disorder: a cross-sectional study in the general Danish population. Denmark [34].**	To examine the relationships between stigma and support for others’ help-seeking behavior regarding alcohol-use disorder as well as to investigate potential gender differences.	Cross-sectional study.	2895 individuals: 1535 male (53.0%) and 1360 female (47.0%). 1427 individuals (49.3%) were aged between 50 and 65 years.	Alcohol-use disorder.	The questionnaire included sections on demographic data, alcohol-related issues, the help-seeking process, and other relevant areas. Difference, Disdain and Blame Scales for Public Stigma questionnaire.	Lower levels of stigma seem to be linked to a higher likelihood of adopting an active support strategy. However, stigma levels were not related to uncertainty about what to say or do, or to sharing concerns with others. Gender differences were minimal: for men, higher stigma levels were linked to a greater likelihood of “avoidance”, whereas for women, lower stigma levels were associated with a reduced probability of “avoidance”.
**Younesi et al. (2023). Crippled with Remorse and Judgment of Others: A Phenomenological Study of Suicide Attempts in Men Dealing with Substance Use. Iran [29].**	To carry out a phenomenological study on suicide attempts among men with substance use problems.	Qualitative design. Semi-structured interview.	12 men (with a history of substance use and suicide attempts).	Substance-related disorders.	Semi-structured interview (including exploratory questions including experiences with suicide attempts, thoughts and feelings related to the suicide attempt, the personal meaning of suicide, etc.).	Ineffective personality patterns and the stigma related to addiction contribute to suicide attempts among men with substance use issues, and they remain at high risk of further attempts if these conditions persist. However, men typically did not seek help for this reason.
**Kumar et al. (2022). Stigma towards dependent drinking and its role on caregiving burden: A qualitative study from Goa, India. India [25].**	To investigate the connection between stigma and caregiving within the context of alcohol-use disorders.	Qualitative design. In-depth interviews.	36 individuals: 11 men with probable alcohol dependence (mean age of 40 years); 12 caregivers of men with alcohol dependence who were not part of the main sample (mean age of 45 years); and 13 general physicians from primary health centers who regularly interacted with individuals with dependent drinking (mean age of 34 years).	Alcohol-use disorder.	Screening: AUDIT. In-depth interviews (including participants’ perceptions and understandings of alcohol-use disorder including its causes and effects, coping mechanisms, help-seeking behaviors, treatment experiences, unaddressed treatment needs, preferred treatment options, and desired treatment outcomes, among other aspects).	Men with likely alcohol dependence, along with practitioners and informal caregivers, displayed some level of misinformation about alcohol dependence. This stigma may have led to short-term, symptom-focused approaches to seeking treatment and delivering interventions by both men with dependence and healthcare providers. It could have resulted in delays in caregivers seeking help and created a challenging environment at home for men recovering from dependence.
**Bhad et al. (2020). A study of pathways to care among opioid-dependent individuals seeking treatment at a community de-addiction clinic in India. India [30].**	To explore the routes to care for individuals dependent on opioids who are seeking treatment at a community-based addiction clinic in India.	Cross-sectional observational study.	100 treatment-seeking drug men (age range: 18–60 years).	Opioid-use disorder.	Semi-structured interviews were developed according to patient self-report (including educational background, employment situation, current housing conditions, and patterns of substance use, among other factors).	Men: insufficient help-seeking behavior and inadequate use of the available addiction services.
**Choi et al. (2017). Older adults who use or have used marijuana: Help-seeking for marijuana and other substance use problems. United States of America (USA) [32].**	To investigate the relationships: (1) between seeking help for marijuana use and the presence of other substance use or mental health disorders, and (2) between seeking help for other substance-use disorders and marijuana use among adults aged 50 and older.	Cross-sectional study.	14.715 individuals (46.73% men).	Marijuana disorder.	DSM-5 to explore marijuana disorder and mental disorders. Information on help-seeking behaviors for substance use problems and/or mental disorders as well as sociodemographic characteristics.	Men with marijuana-use disorders were more inclined to seek assistance for alcohol-related issues, although were less prone to pursue help for nicotine-related troubles.
**Håkansson et al. (2017). Who Seeks Treatment When Medicine Opens the Door to Pathological Gambling Patients-Psychiatric Comorbidity and Heavy Predominance of Online Gambling. Sweden [36].**	To analyze patient characteristics within a new healthcare-based treatment approach for pathological gambling, with an emphasis on exploring potential correlations between types of gambling, psychiatric comorbidities, and gender.	Retrospective review of patient charts from an outpatient facility.	106 individuals: 85 men and 21 women (mean age of 31.5 years).	Gambling disorder.	Retrospective review of patient charts from an outpatient facility (including data on age, gender, method of accessing the facility, reported problematic gambling types, primary type of gambling, and diagnosed psychiatric disorders).	Men were more prone to seeking help compared to women, who experienced a more rapid progression of gambling disorder.
**Mao & Bottorff. (2017). A Qualitative Study on Unassisted Smoking Cessation Among Chinese Canadian Immigrants. China [26].**	To explore how Chinese Canadian immigrant men who smoke view smoking cessation aids and services, and how they use these resources to help them quit smoking.	Qualitative design. Semi-structured interview.	22 men (mean age = 38, SD = 5.0).	Tobacco disorder.	Semi-structured interview (including smoking habits, factors contributing to successful smoking cessation, obstacles hindering smoking cessation efforts, the use of smoking cessation assistance, differences in the pursuit of cessation assistance between Chinese and Canadian smokers, and possible reasons for these differences, etc.).	Chinese immigrant men’s hesitance to use smoking cessation resources arises from their status as immigrants and deeply rooted cultural values of self-control and self-reliance associated with masculinity.
**Meshberg-Cohen et al. (2017). Relationship between substance use and attitudes towards seeking professional psychological help among veterans filing PTSD claims. United States of America (USA) [33].**	To examine attitudes toward treatment among veterans diagnosed with posttraumatic stress disorder (PTSD) including those with and without co-occurring substance-use disorders.	Cross-sectional study.	143 men: (mean age of 33.9 years).	Substance-use disorders.	CAPS. SCID. ATSPPH-SF.	Veterans with comorbid substance-use disorders demonstrated notably less positive attitudes toward seeking help compared to those without this comorbidity. Veterans with substance-use disorders perceived the treatment to be less effective.
**Parkman et al. (2017). How Do People Who Frequently Attend Emergency Departments for Alcohol-Related Reasons Use, View, and Experience Specialist Addiction Services? United Kingdom [27].**	To examine how individuals who frequently visit emergency departments for alcohol-related problems use, perceive, and experience specialized addiction services.	Qualitative design. Semi-structured interview.	30 individuals: 18 men and 12 women (mean age of 47.9 years).	Alcohol-use disorder.	Semi-structured interview (including sociodemographic profiles, specialized addiction services accessed by participants; their perceptions of these services; the reasons they provided for not seeking specialized addiction support; and the forms of treatment or assistance participants indicated they would prefer for managing their alcohol consumption, among other factors).	Women were more prone to seeking support from specialist addiction services than men.
**Stein et al. (2016). Gender Differences in the Life Concerns of Persons Seeking Alcohol Detoxification. United States of America (USA) [31].**	To explore the life worries of individuals seeking alcohol detoxification, a group encountering various life and psychosocial difficulties.	Cross-sectional study.	189 individuals: 138 men and 51 women (mean age of 43.5 years).	Alcohol-use disorder.	Structured interview (apprehension about their alcohol consumption, concerns related to smoking, and anxiety surrounding drug use, among other issues)	Men seeking alcohol detoxification expressed higher levels of worry compared to women in over half of the assessed questions.
**Baxter et al. (2015). Gender differences in felt stigma and barriers to help-seeking for problem gambling. Canada [37].**	To gain insight into how internalized stigma acts as a barrier to seeking help for both men and women dealing with problem gambling.	Concept mapping: a mixed methods approach to participatory research.	28 individuals: 10 men and 18 women (mean age of 53 years).	Gambling disorder.	SOGS-SF. Concept mapping groups: involved four brainstorming sessions, each tailored to a specific participant group: male and female gamblers health care professionals, and individuals with relatives with gambling disorders.	Men and women saw the shame linked to gambling-related financial problems as a major barrier to seeking help. In the case of men, the stigma was related to the addictive nature of gambling and the emotional reactions it provoked. However, women identified several factors as obstacles to seeking assistance including the appealing sensory experience of the gambling environment, denial of their addiction, belief related to luck and the possibility of overcoming the casino as well as the shame associated with dishonest behavior.
**Smith et al. (2015). Does gender moderate the subjective measurement and structural paths in behavioral and cognitive aspects of gambling disorder in treatment-seeking adults. Australia [35].**	To conduct a retrospective assessment of how gender influences structural pathways in the behavioral and cognitive dimensions of gambling disorder, using self-report measures.	Quantitative study. Retrospective design.	454 individuals: 280 men (mean age = 37.4 years, SD = 11.4) and 174 women (mean age = 48.7 years, SD = 12.9).	Gambling disorder.	PGSI. GUS. GRCS.	Men seeking treatment had comparable understandings to women regarding underlying factors such as the severity of problem gambling, gambling-related urges, interpretive bias, and expectancies associated with gambling. However, men reported significantly higher levels of gambling-related urges and interpretive bias in comparison with women, which influenced their behavior in seeking treatment.
**Hanpatchaiyakul et al. (2014). Thai men’s experiences of alcohol addiction and treatment. Thailand [28].**	To examine men’s experiences with the advantages and disadvantages of alcohol consumption to identify the barriers Thai men face regarding alcohol addiction and their decisions to quit drinking.	Qualitative design. Thematic interviews.	13 men (age range: 32–49 years).	Alcohol-use disorder.	Thematic interviews focused on experiences related to alcohol consumption and alcohol treatment.	Three categories of experiences were identified concerning the development of addiction in men: healing the body, drinking as a means of compensation and enhancement for work, and alcohol becoming a constant companion.

**Notes:** Age data varies depending on the information provided by each study. Mean and standard deviation are available only for quantitative studies. The main results have been underlined. **Abbreviations:** ATSPPH-SF: Attitudes Toward Seeking Professional Psychological Help Scale-Short Form; AUDIT: Alcohol-Use Disorder Identification Test; BAC: blood alcohol content; CAPS: Clinician-Administered PTSD Scale; DSM-5: Diagnostic and Statistical Manual of Mental Disorders, 5th edition; GRCS: Gambling Related Cognitions Scale; GUS: Gambling Urge Scale; PGSI: Problem Gambling Severity Index; PTSD: Posttraumatic Stress Disorder; SCID: Structured Clinical Interview for DSM-IV-TR Axis I Disorders, Research Version, Patient Edition; SD: standard deviation; SOGS-SF: South Oaks Gambling Screen Short Form.

### 3.2. The Process of Help-Seeking among Men with Addiction

#### 3.2.1. The Help-Seeking Process from a Gender Perspective

In the context of gambling, both men and women viewed the shame related to financial difficulties caused by gambling as a significant obstacle to seeking assistance [37]. For men, these stigma-related barriers were linked to the addictive nature of gambling and the emotional reactions it provoked [37]. For women, the challenges included the enticing nature of the gambling environment, the negation of their addiction, reliance on luck, the belief that they could outsmart the casino, and the shame associated with dishonest behavior [37].

Men experienced shame at the prospect of acknowledging emotional vulnerability such as using gambling as a coping mechanism, while women felt ashamed of admitting they were drawn in by the allure of the casino and held irrational beliefs about defeating it [37]. Håkansson et al. [36] found that most treatment-seeking patients were male. Although gambling is generally more prevalent among men, the process from gambling initiation to problem gambling may occur more rapidly in women. Furthermore, the interval between the onset of problem gambling and the pursuit of treatment tends to be shorter in women [36]. Furthermore, men reported significantly higher levels of both gambling-related urge and interpretive bias in comparison with women, which influenced their treatment-seeking behavior [35].

Women with alcohol-use disorder were generally more inclined than men to seek and receive support from specialized services. Furthermore, women seemed more likely to seek alcohol-specific support and were less prone to have social support, remunerated or voluntary employment, or go to the gym. Regarding barriers to not attending specialist addiction services, men were more prone than women to report not wanting or needing help, being unaware of available specialized services or support, or encountering mobility issues when accessing these services [27]. Additionally, Stein et al. [31] found that men undergoing alcohol detoxification expressed greater concerns than women in over half of the evaluated issues such as alcohol-related issues, mental health, cigarette smoking, economic difficulties, interpersonal problems, drug use, serious physical diseases, health insurance, sexually transmitted diseases, and community safety. This observation suggests that men might have fewer resources to address these life challenges [31].

In the domain of alcohol-use disorder, women seemed to be less impacted by stigma than men [34]. For men, a higher perception of stigma was strongly correlated with a greater tendency to use avoidance strategies, whereas a lower perception of stigma was related to a decreased likelihood of using such strategies [34]. Conversely, for women, lower levels of perceived stigma were significantly related to a reduced likelihood of adopting avoidance strategies [34]. Avoidance behaviors and the tendency to socially distance oneself from individuals with alcohol-use disorder can be considered as both consequences of stigma and as elements of the stigma process [38]. Men most commonly reported that their experiences in seeking help were preceded by adverse social consequences and considered their overall help-seeking experiences as negative [24].

In the study by Jirwe et al. [23] that focused on older adults with alcohol-use disorder, it was found that after recognizing their problematic alcohol consumption, the subsequent step of seeking help triggered different emotional responses in men and women. All participants highlighted the importance of help-seeking locations that acknowledged their preferences and treated them with dignity. However, the men emphasized that the identity of the person providing help was more important than the physical location of the service. Additionally, the men discussed how, throughout their lives, alcohol dependence had often been viewed as a moral failing or a character defect instead of an illness, which shaped their belief that problematic alcohol use was inherently shameful. In contrast, women associated the stigma with their biological gender and the societal expectation that they should assume a higher level of responsibility for childcare and relatives, which was perceived as conflicting with alcohol use [23].

Regarding tobacco, the main obstacles to accessing tobacco cessation services were identified as misunderstandings about nicotine addiction, negative views on the effectiveness of cessation programs, overconfidence in one’s ability to quit independently, concerns and preferences about the characteristics of healthcare providers at clinics, a male-oriented culture that deterred men from seeking assistance, the social stigma associated with tobacco use by women in Arab societies, and the shortage of time to go to addiction clinics [22].

#### 3.2.2. Barriers and Facilitators in the Help-Seeking Process among Men with Addiction

In men with gambling disorders, the primary barriers to seeking help included: (1) the perception that the economic consequences of gambling create stigma and act as a barrier to seeking assistance; (2) the tendency to use gambling as an escape from reality, wherein individuals may employ excuses or irrational justifications to gamble as a means of alleviating worries and finding solace; (3) stigma-related barriers linked to emotional responses to gambling such as loss of self-respect, frustration from losses, feelings of personal failure, shame, desperation, and emotional distress including anxiety and suicidal thoughts; and (4) stigma-related barriers associated with family reactions to the severity of the addiction including interpersonal conflicts, family anger, marital dissolution, and dishonesty [37].

In men with alcohol-use disorder, higher stigma was significantly related to greater possibilities of employing an avoidance strategy, while there was a tendency suggesting that lower stigma correlated with a reduced likelihood of avoidance behavior [34]. Furthermore, lower stigma was linked to an increased likelihood of seeking help online [34]. Other identified barriers in the help-seeking process included negative beliefs and attitudes associated with seeking help, which are closely linked to gender roles. Additionally, the perception that treatment is a financial matter rather than a personal one, a lack of respect or understanding from professionals, and the presence of stigma among these professionals also impeded help-seeking [24]. Shame and low self-esteem may converge to form another significant barrier to the treatment of alcohol addiction [28]. Furthermore, additional barriers to help-seeking in people with alcohol-use disorder include the tendency of men to focus primarily on physical problems rather than the social or psychological issues of their addiction. Emphasizing physical well-being over addictive behavior, consuming alcohol during their working days, and the effects of self-stigmatization and feelings of shame in treatment or treatment attempts further exacerbate these barriers [28]. The lack of awareness about available specialist addiction services and the types of support they offer, along with mobility issues (i.e., health problems), have emerged as significant barriers to addressing alcohol-use disorder [27].

In the context of tobacco, another key barrier to seeking cessation support is the masculine culture that deters men from pursuing help [22,26]. This culture frames smoking as a symbol of masculine strength, manhood, and personal freedom, thereby deterring men from seeking assistance to quit [22,26]. Additional barriers identified include practical challenges such as limited access to information on smoking cessation and difficulties in obtaining cessation services as well as cultural obstacles like the denial of nicotine addiction, distrust in the efficacy of cessation aids, a preference for self-reliance in problem-solving, and privacy concerns regarding the use of cessation services [26]. Furthermore, Choi et al. [26] found that men with marijuana-use disorder were more inclined to seek help for alcohol-related issues, however, were less prone to seek assistance for nicotine-related disorders.

Bhad et al. [30] pointed out that in the context of opioid-use disorder, men tended not to seek help and exhibited a poor utilization of the available services. The primary source of initial help-seeking suggestions was friends (71%), followed by family members or neighbors (27%), with self-referral being rare (2%) [30]. The median delay before the first attempt to seek treatment was 9.5 years.

Referring to substance-use disorders, Meshberg-Cohen et al. [33] indicated that veterans with co-occurring substance-use disorders demonstrated significantly less favorable attitudes toward seeking help compared to those without this comorbidity. Additionally, the perceived value of treatment was notably lower among veterans with substance-use disorders.

Regarding possible facilitators in the help-seeking process, social support emerged as one of the main factors in people with alcohol-use disorder [34]. Social support is a key factor in promoting both mental and physical health, especially when individuals face stressors [39]. Support from others can help mitigate stress and might also boost self-efficacy [40]. For individuals undergoing addiction treatment, greater perceived social support from relatives, friends, and other important people for the individual is related to reduced levels of internalized stigma, suggesting that social support might act as a protective factor against self-stigma [41]. Moreover, social support has emerged as a critical factor in encouraging help-seeking behaviors among those with addiction, aiding them in initiating treatment [34,42,43,44]. The authors also enhanced the role of social networks in promoting a higher use of addiction clinics and ameliorating help-seeking behaviors in individuals with opioid-use disorder [30].

#### 3.2.3. Potential Research Gaps in the Field of Intervention and Research

The primary gaps identified in this systematic review were the lack of, or deficiencies in, incorporating a gender perspective in the existing research. Many studies failed to report data separately by gender or to include gender as a category in analysis and intervention. This omission is particularly critical in the context of addiction, where evidence indicates that women and men exhibit different patterns of behavior [37].

Additionally, there is a lack of interventions focused on eliminating or decreasing social stigma and obstacles to help-seeking, particularly within the male population. Some authors of the analyzed studies suggest potential treatments that should be considered.

Baxter et al. [37] point out that peer outreach and support represent a promising strategy for increasing the engagement of male gamblers in treatment. Individuals with lived experience may effectively address stigma and connect with men experiencing gambling problems, thereby facilitating their engagement in treatment for mental health concerns. Participants in Gambler’s Anonymous are significantly more inclined to modify their gambling behaviors and seek professional assistance compared to those who are not involved in these groups [45]. Furthermore, individuals who received treatment for problem gambling and had prior participation in anonymous support groups are more likely to achieve success compared to those who did not attend these groups [46]. Anonymous groups can enhance personal awareness of the risks related to gambling, and peers in the anonymous groups’ self-help model play an educational role, aiding individuals in overcoming stigma and pursuing treatment for their issues [37].

Additionally, the widespread occurrence of online gambling and its frequent association with psychiatric comorbidities underscores the unmet treatment needs of problem gamblers. This problem arises in a setting in which treatment resources are still insufficient, while gamblers are consistently exposed to online gambling promotions through several media channels. Effective treatment for pathological gambling may require a focused approach to addressing psychiatric comorbidities, with particular attention to female clients [36]. A more comprehensive understanding of how gender moderates subjective measurements and structural pathways in the behavioral and cognitive patterns of gambling disorder among treatment-seeking in the adult population is crucial for the development of treatment strategies including relapse prevention [35].

Wallhed Finn et al. [34] indicated that there are two interconnected and crucial areas of intervention in individuals with alcohol-use disorder: first, the necessity to decrease the stigma related to alcohol-use disorder, and second, the role of social support in encouraging help-seeking for alcohol-use disorder. Moreover, given the high prevalence of suicide between individuals with substance abuse as well as the connection between stigma, addiction, and suicide attempts, it is crucial for society, particularly health professionals, to remain vigilant in order to prevent and reduce suicide in this population [29].

On purpose, Kumar et al. [25] highlighted that stigma serves as a significant barrier to effective treatment and care, while also negatively impacting the caregivers’ mental health and decision-making in caregiving. The stigma associated with alcohol dependence, expressed through ignorance, prejudice, and discrimination, is pervasive in households, workplaces, and healthcare providers. This stigma can worsen the caregiving burden on relatives [25]. Implementing policies, educational programs, and campaigns focused on reducing these forms of stigma could improve access to more inclusive and suitable healthcare services, enhance the well-being of family caregivers, and lead to better treatment results for individuals with alcohol dependence [25]. Additionally, Parkman et al. [27] suggested that case management and assertive approaches seem to be effective approaches to address treatment in this population, especially for men with alcohol-use disorder.

Men’s narratives around drinking culture and its association with male bonding in homosocial group settings represent a notable obstacle to seeking alcohol treatment and maintaining abstinence in certain groups. This challenge emphasizes the relevance of addressing masculinity and dominant ideals to reduce their influence at all stages of treatment for men including those with alcohol addiction [24,28] and those with tobacco dependence [26].

It has also been recommended that individuals with alcohol dependence receive person-centered treatments to enhance self-adherence and treatment efficacy [23]. On purpose, recognizing the daily concerns of individuals seeking inpatient alcohol detoxification can help providers personalize their services to better align with their patients’ life contexts [31]. Addressing critical issues including mental health, economic difficulties, interpersonal problems, and other substance use may impact the effectiveness of detoxification services and inform decisions regarding aftercare treatment [31].

Furthermore, it is essential to enhance the availability, accessibility, and awareness of treatment options to decrease delays in seeking treatment for individuals with opioid-use disorder [30]. In addition, pathways to care seem to be a crucial approach for examining the patients’ help-seeking behavior [30]. Its effectiveness in understanding treatment delays, developing efficient referral systems, and informing policy decisions has been well-established [30].

In general, the authors agree on the need for a better comprehension of the barriers in the help-seeking process to implement more effective strategies [22,23,25,30,34,37].

### 3.3. Quality of Selected Studies and Risk of Bias

According to the criteria of the Effective Public Health Practice Project Quality Assessment Tool (EPHPP) [21], the quality of the analyzed studies was weak in all cases [22,23,24,25,26,27,28,29,30,31,32,33,34,35,36,37].

Studies have indicated several limitations that should be solved in future research to overcome existing shortcomings. The main limitations were: the use of self-report measures [31,32,34], the cross-sectional design not allowing for concluding causality [34], the lack of similar studies to establish comparisons and better hypothesis [27], limitations referring to the methodological and/or theoretical approach [25,26,29,30,31,32,33,35,36], the high number of analyses [34], difficulties in the access to female participants [22,30], difficulties in generalizing the results arising from the qualitative methodology and the specific characteristics of the sample [24], and limitations derived from the sample characteristics and/or the number of participants [22,23,24,25,26,27,29,30,31,32,33,35,36].

Additionally, this systematic review identified limitations that were not explicitly mentioned by the authors including the following: failure to acknowledge the study’s limitations [37], insufficient data on sample characteristics [27,29,32,37] and/or the type of addiction [29], lack of procedural details [30,31,34], the omission of specific methods or study design [31,32,33,35], and the lack of strategies to ensure the quality of the qualitative studies [22,23,24,25,26,27] in accordance with Guba’s recommendations [47], the Standards for Reporting Qualitative Research (SRQR) [48], and the guidelines of the Consolidated Criteria for Reporting Qualitative Studies (COREQ) [49].

**Table 2:** Quality of selected studies and risk of bias.

First Author (Publication Year)	Selection Bias	Study Design	Confounders	Blinding	Data Collection Method	Withdrawals and Dropouts	Global Assessment	Additional Biases
Al-Jindi et al. (2024) [22].	M	S	W	W	S	W	W	Difficulties in the access to female participants due to the cultural taboos related to smoking, particularly within Qatari culture. Small sample size concerning former smokers.
Jirwe et al. (2024) [23].	W	S	W	W	S	M	W	Small sample size. The study only included individuals seeking treatment and did not explore those who were not seeking treatment, who may have different perceptions that require further investigation. Lack of strategies to ensure the quality of the qualitative studies.
Robles et al. (2024) [24].	W	S	W	W	S	W	W	The findings may vary significantly among men of Mexican ethnicity in populations beyond the USA/Mexico border and do not adequately capture the diverse experiences of men of Mexican ethnicity as a whole. Difficulties in generalizing the results arise due to the qualitative methodology.
Wallhed Finn et al. (2023) [34].	M	M	W	W	M	W	W	Use of self-report measures. The high number of analyses. Lack of procedural details. Limitations due to the cross-sectional design (establishing causality was not possible).
Younesi et al. (2023) [29].	W	S	W	W	S	W	W	Small sample size. Insufficient data on sample characteristics. Insufficient data on the type of addiction. Difficulties in generalizing the results arising from the qualitative methodology.
Kumar et al. (2022) [25].	W	S	W	W	M	W	W	Did not include women in the sample. Did not explore the category of stigma deeper. Did not delve deeper into elements such as the participants’ socioeconomic status and its influence on the results. Difficulties in generalizing the results arising from the qualitative methodology. The lack of strategies to ensure the quality of the qualitative studies.
Bhad et al. (2020) [30].	W	M	W	W	M	W	W	Difficulties in the access to female participants. Lack of procedural details. The WHO encounter form used in this study was not translated into the local language. Did not include data on non-dependent use of other psychoactive substances and their effects on pathways to care. Difficulties in generalizing the results. Limitations due to the cross-sectional design (establishing causality is not possible).
Choi et al. (2017) [32].	M	S	M	W	S	W	W	Omission of specific methods or study design. Use of self-report measures. Did not include data on treatment duration and adherence, treatment initiation, lifetime help-seeking, and the onset of marijuana and other substance-use problems/disorders as well as mental disorders. Did not analyze each type of treatment included in the help-seeking process due to the small sample size. Did not conduct subgroup analyses due to the small sample size. The potential negative effects of social desirability and recall bias regarding an event that occurred a long time ago. Limitations due to the cross-sectional design (establishing causality is not possible).
Håkansson et al. (2017) [36].	M	S	W	W	S	W	W	Established a short period for data collection, which made the analysis difficult and limited the sample size. Insufficient data on sample characteristics (e.g., patients’ duration of problem gambling, the extent of their gambling behavior, including the amount of money spent, etc.). Did not include historical diagnoses in the comorbid psychiatric disorders.
Mao & Bottorff. (2017) [26].	W	S	W	W	S	W	W	Limitations associated with the recruitment method. Difficulties in generalizing the results arising from the qualitative methodology. The lack of strategies to ensure the quality of the qualitative studies.
Meshberg-Cohen et al. (2017) [33].	W	M	M	W	S	M	W	Omission of specific methods or study design. Did not include treatment history. Limitations due to the cross-sectional design (establishing causality is not possible).
Parkman et al. (2017) [27].	W	S	W	W	S	W	W	Limitations associated with the recruitment method. The lack of similar studies. Small sample size. Insufficient data on sample characteristics. Difficulties in generalizing the results arising from the qualitative methodology. The lack of strategies to ensure the quality of qualitative studies.
Stein et al. (2016) [31].	W	S	W	W	S	M	W	Omission of specific methods or study design. Limitations associated with the recruitment method. Use of self-report measures. Lack of procedural details. Lack of objective data (e.g., from health records) concerning the participants’ mental and physical health diagnoses, personal finances, or social support. Did not include the perceived treatment needs. Did not explore the possible association between concerns and the specific care received. Limitations due to the cross-sectional design (establishing causality is not possible).
Baxter et al. (2015) [37].	W	S	W	W	S	M	W	Failure to acknowledge the study’s limitations. Small sample size. Insufficient data on sample characteristics.
Smith et al. (2015) [35].	M	S	M	W	S	W	W	Omission of specific methods or study design. Failure to gather data concerning formal evaluations of comorbid disorders. The findings cannot be generalized to a wider array of subpopulations of gamblers.
Hanpatchaiyakul et al. (2014) [28].	W	S	W	W	S	M	W	Small sample size. Lack of strategies to ensure the quality of the qualitative studies.

**Notes:** Effective Public Health Practice Project Quality Assessment Tool (EPHPP) [21]. S = Strong (no weak ratings), M = Moderate (one weak rating), or W = Weak (two or more weak ratings). **Abbreviations:** USA: United States of America.

## 4. Discussion

The help-seeking process in the male population with addiction problems has emerged as a relevant field in both research and clinical practice due to its significance. What contributions does this systematic review provide regarding this topic? The previous results pointed out how gender differences influence help-seeking behavior, with women generally exhibiting a higher propensity to seek assistance for addiction-related issues than men. Men with addiction represent a significant target for intervention in health and social contexts, requiring a more specific and personalized approach to address their needs and enhance the help-seeking process. Moreover, seeking help for addiction—whether related to substance use or gambling—can be hindered by several barriers for men. In this section, a detailed analysis will be conducted regarding these matters.

Similar to mental illness, help-seeking in the context of addiction including addiction to substances (i.e., alcohol, tobacco, opioids, marijuana) and gambling might be hindered by public stigma and discrimination [22,23,25,34,37,50]. The reluctance to seek help may be influenced by concerns about self-identity and reputation in gambling [35,37], alcohol-use disorder [23,25,34], and to a lesser extent in tobacco [22]. Individuals may experience feelings of shame, embarrassment, and guilt if they perceive themselves as failing to meet social expectations due to a lack of control over their behavior and/or the lack of adherence to male gender stereotypes [22,34,37]. It is essential to understand that the construction of masculinity is a continuous process of demonstrations before other males, as it is conferred by other men. This process begins in childhood, as boys must show that they are not feminine [51].

It has been established that initiation, usage patterns, disease progression, and help-seeking behaviors are significantly influenced by gender differences, which are shaped by biological, psychological, cultural, and socioeconomic factors. Considering gender differences, women generally tend to seek help for addiction including gambling [14,36] and alcohol-use disorder [27]. In contrast, men are less inclined to seek assistance for addiction (i.e., gambling, alcohol-use disorder, opioid-use disorder, etc.) due to perceived embarrassment, shame, and the related stigma, among other possible causes [15,27,30,37]. Parkman et al. [27] reported that women with alcohol-use disorder are generally more inclined than men to seek and receive support from specialized services. Similarly, men with alcohol-use disorder commonly reported that their experiences in seeking help were preceded by adverse social consequences and regarded their overall help-seeking experiences as negative [24]. In the context of tobacco use, prevailing male gender norms appear to discourage men from seeking help, while gender stereotypes also negatively impact women by amplifying the negative connotations associated with tobacco use among them [17]. Research also indicates that women are generally more inclined to seek assistance for gambling-related issues [14]. Significant gender differences have been identified in the likelihood of seeking help for problem gambling [15]. Men are often less willing to seek assistance due to feelings of shame, embarrassment, and the associated stigma. Conversely, women may delay seeking help, often due to denial about their problem or reluctance to quit gambling, as this could result in the loss of an important social support network. Men experienced distress due to the stigma surrounding emotional responses to gambling and mental health issues when considering seeking help, while women perceived gambling as an attractive lifestyle choice, which acted as a barrier to seeking assistance [37].

Individuals with gambling disorders may avoid seeking treatment due to the stigma they anticipate. The desire to avoid embarrassment, shame, and social stigma often leads them to forgo seeking assistance. Indeed, this fear of stigma has been identified as a relevant predictor of delayed help-seeking [52]. For example, Hing and Nuske [50] discovered that individuals frequenting gambling establishments were unwilling to seek assistance due to feelings of shame. Additionally, the stigmatization of clients with pathological gambling by professionals, exacerbated by the terminology used by the medical field and the media, constitutes another major barrier [15,53]. Many service providers endorse common stereotypes of pathological gamblers, portraying them as individuals who lack self-control, are irresponsible, and are prone to dishonesty and criminal behavior [15,53]. Such labeling contributes to stigma, which can significantly hinder individuals from seeking help, particularly when problem gambling is seen as socially unacceptable and is associated with secrecy and shame. This issue is further exacerbated by the perception among colleagues and family members that those who engage in problem gambling are considered to be lacking in self-control and irresponsibility [54].

A clear link between public and self-stigma and attitudes toward supporting others in seeking help for addiction has been also reported [23,25,34]. Public stigma is defined by the negative perceptions and stereotypes that the majority holds toward a specific group [11]. Conversely, self-stigma may arise when individuals belonging to a stigmatized group start to internalize the public stigma aimed at them. Perceptions of stigma can negatively affect not only the decision to seek help, but also the timing of that help-seeking behavior [8,9,10]. These stigmas surrounding men with alcohol-use disorder [23,25,34] may affect not only the individuals with addiction themselves, but also family caregivers and healthcare providers [25]. The negative impact of public stigma on addiction is evident in the relationship between public stigma and discrimination [22,23,25,34,37,50]. The findings underscore the relevance of reducing stigma and encouraging supportive behaviors toward others’ pursuit of treatment [25,34]. The stigma related to alcohol-use disorder is recognized as one of the most significant barriers to seeking treatment [25,34,55,56]. Public stigma surrounding addiction is evident in the assignment of negative labels such as reckless, hopeless, helpless, unreliable, dangerous, and insane. This labeling is often rooted in the assumption that individuals actively choose to engage in addictive behaviors and are therefore accountable for their addiction. Such negative perceptions can elicit emotional responses including fear and anger, which may subsequently lead to discriminatory actions such as dehumanization and reduced support from public and healthcare systems. Furthermore, individuals who experience stigma frequently encounter self-stigmatization, resulting in psychological distress that adversely affects their ability to access healthcare services [57].

Moreover, the stigma associated with addiction is a significant factor contributing to suicide attempts [29]. Park and Park [58] suggested that stigma might result in several negative emotional outcomes including diminished self-respect and neglect. To cope with stigma, individuals often withdraw from society, isolate themselves, and refrain from disclosing their issues. Consequently, they may delay seeking help, believing that concealing their problems will make them disappear [59]. Therefore, it is essential to address and prevent suicide among this vulnerable population. Another common barrier to seeking treatment in individuals with alcohol-use disorder is the low recognition of the problem [60].

Moreover, most men evaluated the need for assistance based on their cultural values and the societal expectations of masculinity [24]. Robles et al. [24] found that these men prioritized family commitments over personal needs when considering seeking help. They recognized the need for help when their alcohol use began to risk their ability to fulfill their family responsibilities. These men placed high importance on being role models for their families and children and esteemed the role of a responsible man. Their self-imposed standards often served as a barrier to seeking help, as doing so was viewed as a loss of control and a failure to set a positive example. They experienced a sense of duty to uphold these roles, and in some cases, seeking help was seen as contradictory to their self-expectations as men, with the belief that needing help indicated a lack of strength [24].

Beliefs and attitudes related to help-seeking may also impact the decision-making process for men with alcohol-use disorder [24]. For some men, the need for help was associated with an inability to care for oneself, leading to feelings of shame and failure. Several men articulated that being a “man” involved the capacity for self-reliance [24]. These views underscore the importance placed on independence and self-sufficiency, and the perception that one should be a source of help rather than seeking it [24].

In the differentiated socialization perpetuated by a patriarchal society, not all emotions are repressed. Hegemonic masculinity upholds gender mandates such as “being strong”, which applies not only to the physical realm, but also to the emotional. On the one hand, emotions like tenderness, compassion, fear, sadness, or pain are perceived as signs of weakness in men. On the other hand, aggression, anger, and indifference—emotions associated with action and initiative—are regarded as traits that facilitate a man’s acceptance within his peer group [61]. The primary gender mandates are “being strong” and “being brave”.

To better understand the relevance of gender norms related to masculinity and their influence on addictions and help-seeking [22,23,24,26,28], it is essential to highlight the ideas proposed by Martínez and Luján [62]. This study outlined several fundamental characteristics of masculine gender mandates that affect addictions including physical strength, the constant demonstration of “manhood”, and the perception of emotions and their expression as signs of vulnerability (e.g., “a real man never cries”). Furthermore, it addressed the mandate to “be tough” (i.e., to avoid seeking help) and emphasized aggression and anger as key emotions [62]. These gender norms position risk-taking as a value of masculinity, rendering fear an emotion that men should not display, as it can lead them to perceive themselves as weak and vulnerable.

In men with addiction issues, particularly during adolescence, many abusive substance-use behaviors are often associated with peer validation in the demonstration of “manhood”, linked to risk-taking actions involving substance use.

Additionally, it is noteworthy that in most rehabilitation processes for alcohol and other substances as well as in gambling, recovery involves reconnecting with emotions. In fact, not only is recognizing emotions important, but expressing them is equally vital. These therapeutic tools are fundamental, as the success of recovery for many individuals hinges on the acknowledgment and expression of fear, pain, shame, and failure—factors that significantly influence men’s desire to rehabilitate.

In alignment with previous findings, the conformity to norms traditionally associated with masculine roles (i.e., dominance, womanizing, aggressiveness, and risk-taking behaviors) has been positively associated with increased alcohol consumption [6]. In addition, some authors have underscored the necessity of addressing concepts of masculinity and associated hegemonic ideas to mitigate the barriers faced by men with alcohol addiction in accessing treatment and ceasing alcohol consumption [28]. Risk-taking contributes to the construction of masculine identities, and it is a way to share stories related to drinking that involve risk-taking, which is crucial in forming and sustaining friendships between men [63,64,65]. Masculinity, therefore, is understood as a set of mechanisms that aid men in constructing their identity. This identity necessitates actions that continuously demonstrate and affirm that men are “real” men. Any indication that might suggest otherwise regarding their masculinity can negatively impact how their manhood is perceived by other men [51]. The experiences of men highlight the pressures of masculine culture, especially the expectations to be the primary breadwinner and to sustain employment, as discussed by Connell and Messerschmidt [66]. Hegemonic notions of masculinity appear to play a relevant role in alcohol addiction, and an understanding of how gender is performed should be incorporated when healthcare services develop appropriate treatment approaches [28].

It is essential to examine the extent to which men who experience addictions do so as a result of negative experiences associated with the enactment of hegemonic masculinity. Ranea [51] notes that mental health issues among men have risen, particularly evident during recent economic crises, during which many men lost their jobs. This disruption affected their role as family providers, influencing their perception of failing to fulfill a key mandate of hegemonic masculinity, which, in many cases, led to existential and emotional crises. This phenomenon is also reflected in another influential gender mandate: “being strong” [51]. As previously discussed, this mandate is linked to the reluctance to seek help when necessary.

Indeed, gender stereotypes appear to endure throughout life, as demonstrated by the study by Jirwe et al. [23]. In this research, older men attributed their loss of control over alcohol consumption to changes in life circumstances such as decreased responsibilities and a reduced sense of belonging. Alcohol dependence was closely related to feelings of shame and stigma. These men associated their shame with a perceived lack of personal character, noting that in their generation and upbringing, alcohol dependence was seen as a character flaw. Their inability to control their drinking thus led to feelings of shame.

Regarding tobacco, masculine culture was considered as a relevant barrier to seeking addiction services between university students, whereas cultural attitudes that glorify smoking created social pressure on men [22,26]. Participants noted that their peers often discouraged them from quitting, viewing the pursuit of help-seeking as a sign of weakness [22]. They acknowledged that in their culture, smoking was viewed as a masculine activity, and seeking assistance from cessation services frequently resulted in stigmatization [22]. As a result, they preferred not to disclose their use of these services to avoid being seen with “pity”. Some participants also believed that their friends would respond to their seeking cessation services with skepticism such as asking, “Why would you go to a doctor for that? You can just quit on your own” [22].

Similarly, Mao et al. [26] identified two main categories of barriers to seeking assistance for tobacco dependence: practical barriers and cultural barriers. Practical barriers comprised a lack of enough information about smoking cessation resources and problems with attending these services. Cultural barriers encompassed the denial of nicotine addiction, skepticism about the effectiveness of cessation aids, a preference for self-reliance in solving problems, and privacy concerns when using cessation services. The study found that Chinese immigrants’ hesitance to seek smoking cessation support was influenced by both their status as immigrants and culturally ingrained values of self-control and self-reliance [26]. This highlights the detrimental effects of male gender norms and underscores the need to address the intersectional factors within addiction treatment.

Additional barriers to seeking help for tobacco disorders include concerns about being judged, fear of failure, a perceived lack of information regarding available resources, and the belief that these services are costly [67,68,69]. Morphett et al. [70] also identified several other obstacles such as challenges in managing symptoms associated with nicotine withdrawal, the culture of cigarette in which sharing a cigarette is part of the friendship codes, biases and/or myths like the belief that the determination alone ensures successful process to quit the tobacco habit, and the belief that not smoking might be detrimental, especially after previous unsuccessful attempts with assisted cessation. Further barriers in the process of seeking help for tobacco cessation include difficulties encompassing managing stress and emotional regulation, deriving pleasure from smoking, nicotine dependence, boredom, inadequate support from the healthcare system, limited access to cessation services, societal acceptance and availability of smoking, concerns about weight gain, previous unsuccessful attempts at cessation, attitudes toward addiction treatments, low perceived risk of harm, lack of motivation, and cultural values that emphasize self-reliance [71,72].

Choi et al. [32] found that in the context of marijuana use, individuals with concurrent drug or nicotine-use disorders were nearly three times more prone to seeking help. Conversely, alcohol-use disorders and mental health disorders did not correlate with increased help-seeking for marijuana-related disorders. The study highlighted a lower use of treatment options between those with marijuana-use disorder, underscoring the necessity to identify and address both individual and systemic barriers to accessing treatment [32]. It is essential to continue researching the impact of comorbidities on the help-seeking process.

Bhad et al. [30] found that men with opioid-use disorder often avoided seeking help and showed inadequate use of the available services. Furthermore, friends and family members played a crucial role in initiating the help-seeking process [30]. This further underscores the relevance of social support in the help-seeking process.

Similarly, Meshberg-Cohen et al. [33] reported that veterans with co-occurring substance-use disorders exhibited lower favorable attitudes toward seeking help than those without such comorbidities. Furthermore, these veterans perceived the value of treatment to be markedly lower. This represents further evidence of the negative impact of addiction on the perception of treatment, and consequently on the low rate of help-seeking.

It is noteworthy that the negative impact of gender stereotypes on men with addictions persists across different cultures and types of addictions. This suggests that it is a common pattern, albeit with varying manifestations depending on cultural context and specific sample characteristics.

The main limitation of the current research is associated with the weakness in the gender analyses of available studies and the scarce scientific evidence focused on the male sample. In some cases, the results were not presented separately for each gender. Consequently, it was not possible to discuss the data from a gender perspective in the discussion, limiting the scope of the review. It is essential to conduct further research on the help-seeking process in men, ensuring both theoretical and methodological rigor to develop stronger scientific evidence in this field. These improvements in the research area will have a direct and positive impact on clinical practice. Moreover, it is necessary to incorporate a gender perspective as part of the basis of our research to address the need for a better and more personalized understanding and treatment of men with addiction. The principal strength of the current systematic review lies in its novelty as well as the clinical and social implications.

Future research should involve a more in-depth exploration of the beliefs, values, and experiences of individuals with addiction, their relatives, and health and social professionals, with particular emphasis on men, who are often underrepresented in this context. Additionally, there is a need to develop culturally adapted intervention strategies based on the biopsychosocial model, as opposed to the biomedical model, which often focuses exclusively on diagnosis and symptom reduction. It is also necessary to incorporate a gender perspective and conduct a more in-depth analysis of intersectionality within this context.

Crenshaw (1989) [73] defined intersectionality as the phenomenon by which each individual experiences oppression or enjoys privilege based on their membership in multiple social categories. This perspective is essential in the field of addiction, especially when aiming to intervene with individuals currently facing these issues. Beyond gender-related factors, it is crucial to apply this perspective to other variables such as sex, ethnicity, place of origin, social class, educational level, and economic status. All of these variables are vital for understanding how gender has been shaped in individuals with addiction problems and were often inadequately explored in the reviewed studies.

When analyzing difficulties related to addictions, it is essential to consider social class. Being a man in a working-class or impoverished neighborhood differs significantly from being a man in a wealthy one. Similarly, addressing issues related to intimate relationships and sexuality with a man whose culture or education is rooted in sexist and unequal behaviors differs from engaging with a man from a more egalitarian background. Furthermore, age plays a significant role; discussing these topics with a 65-year-old man is not the same as with a 19-year-old. It is also necessary to consider men who do not conform to heteronormative sexuality. Therefore, the analysis of these variables should extend beyond merely including age and place of birth and should provide insights that help develop more diverse and comprehensive intervention strategies for men with addiction problems.

Moreover, it would be beneficial to investigate the help-seeking process for addictions other than alcohol and gambling, which are the most frequently studied. It is also necessary to address the theoretical and methodological limitations identified in the included studies.

Regarding the clinical implications of previous findings, it is essential to develop and evaluate the effectiveness of psychoeducational programs aimed at reducing the social stigma associated with addiction. Such programs could be beneficial for individuals seeking help. Additionally, social and health professionals require further education and training to better identify potential individuals who need help and to avoid reinforcing social stigmas. Similarly, media and social network professionals need training and education on addictions, social stigma, and their role in perpetuating or reducing myths and stereotypes. These stereotypes represent some of the most relevant barriers to seeking help for individuals with addictions, particularly for men. Moreover, it is advisable to develop educational campaigns for the general population to help reduce social stigma and myths associated with addiction and to facilitate the rehabilitation of these individuals. Such campaigns would also serve to increase public awareness about the types of addiction, warning signs, available resources for seeking help, and effective strategies for addressing addiction.

Furthermore, another relevant clinical implication is the need to incorporate a gender perspective in the field of addiction, not only in diagnosis, but also in prevention and treatment processes. As previously discussed, women and men exhibit different patterns of addiction and require different approaches. Furthermore, it is crucial to include men as a distinct category in research and clinical practice to develop more effective and person-centered treatments.

Based on the findings of the reviewed studies and as part of the clinical implications of this systematic review, it is recommended that health professionals: (1) develop interventions that include gender as a core issue in diagnosis, treatment, and prevention; (2) implement strategies to facilitate the help-seeking process in men (e.g., providing more information and developing campaigns to increase the availability, accessibility, and awareness of treatment options); (3) enhance the engagement and self-adherence of men with addiction during their treatment; (4) work to prevent social stigma; and (5) remain vigilant regarding potential suicidal behavior in this population.

## 5. Conclusions

Gender differences play a relevant role in help-seeking behavior, with women generally showing a greater propensity to seek assistance for addiction-related issues compared to men. In addition, men, more so than women, believed that emotions related to addiction (e.g., shame, embarrassment) would prevent people from seeking the help they required.

Similar to mental illness, seeking help for addiction—encompassing substance use and gambling—may be made difficult through several barriers, especially public stigma and discrimination, which seem to be greater in alcohol and gambling compared to tobacco. Other identified barriers in the help-seeking process include negative beliefs and attitudes toward seeking help, which are closely related to male gender roles. Furthermore, the perception of treatment as a financial issue rather than a personal one, a lack of respect or understanding from professionals, and the stigma among these professionals also hinder help-seeking.

We recommend the integration of a gender perspective in addiction diagnosis, prevention, and treatment. As noted, addiction patterns differ between men and women, thereby requiring tailored approaches. It is also essential to consider men as a distinct category in research and clinical practice to develop more effective and personalized treatments. Furthermore, it is necessary to set up possible facilitators of the help-seeking process, in particular, social support for people with addictions.

## Data Availability

The data that support the findings of this study are available on request from the corresponding author.
